# Co-designing drug alerts for health and community workers for an emerging early warning system in Victoria, Australia

**DOI:** 10.1186/s12954-023-00761-6

**Published:** 2023-03-09

**Authors:** Rita Brien, Isabelle Volpe, Jasmin Grigg, Tom Lyons, Caitlin Hughes, Ginny McKinnon, Stephanie Tzanetis, Sione Crawford, Alan Eade, Nicole Lee, Monica J. Barratt

**Affiliations:** 1 Turning Point, Eastern Health Statewide Services, Richmond, Australia; 2 Monash Addiction Research Centre, Eastern Health Clinical School, Monash University, Melbourne, Australia; 3 Social and Global Studies Centre, RMIT University, Melbourne, Australia; 4 Drug Policy Modelling Program, Social Policy Research Centre, UNSW Sydney, Sydney, Australia; 5 Department of Health, State Government of Victoria, Melbourne, Australia; 6 Law and Commerce, Flinders University, Adelaide, Australia; 7 National Drug and Alcohol Research Centre, UNSW, Sydney, Australia; 8 Harm Reduction Victoria (DanceWize), Melbourne, Australia; 9 CanTEST (Direction Health Services, Pill Testing Australia, Canberra Alliance for Harm Minimisation and Advocacy), Canberra, Australia; 10 Safer Care Victoria, Melbourne, Australia; 11 Department of Paramedicine, Monash University, Melbourne, Australia; 12 360Edge, Melbourne, Australia; 13 National Drug Research Institute, Curtin University, Perth, Australia; 14 Digital Ethnography Research Centre, RMIT University, Melbourne, Australia

**Keywords:** Drug alerts, Harm reduction, Emerging drugs, Early warning systems

## Abstract

**Background:**

Alerts about changes in unregulated drug markets may be useful for supporting health and community workers to anticipate, prevent, and respond to unexpected adverse drug events. This study aimed to establish factors influencing the successful design and implementation of drug alerts for use in clinical and community service settings in Victoria, Australia.

**Methods:**

An iterative mixed methods design was used to co-produce drug alert prototypes with practitioners and managers working across various alcohol and other drug services and emergency medicine settings. A quantitative needs-analysis survey (*n* = 184) informed five qualitative co-design workshops (*n* = 31). Alert prototypes were drafted based on findings and tested for utility and acceptability. Applicable constructs from the Consolidated Framework for Implementation Research helped to conceptualise factors that impact successful alert system design.

**Results:**

Timely and reliable alerts about unexpected drug market changes were important to nearly all workers (98%) yet many reported insufficient access to this kind of information (64%). Workers considered themselves ‘conduits’ for information-sharing and valued alerts for increasing exposure to drug market intelligence; facilitating communication about potential threats and trends; and improving capacity for effective responding to drug-related harm. Alerts should be ‘shareable’ across a range of clinical and community settings and audiences. To maximise engagement and impact, alerts must command attention, be easily recognisable, be available on multiple platforms (electronic and printable formats) in varying levels of detail, and be disseminated via appropriate notification mechanisms to meet the needs of diverse stakeholder groups. Three drug alert prototypes (SMS prompt, summary flyer, and a detailed poster) were endorsed by workers as useful for supporting their work responding to unexpected drug-related harms.

**Discussion:**

Alerts informed by coordinated early warning networks that offer close to real-time detection of unexpected substances can provide rapid, evidence-based drug market intelligence to inform preventive and responsive action to drug-related harm. The success of alert systems requires adequate planning and resourcing to support design, implementation, and evaluation, which includes consultation with all relevant audiences to understand how to maximise engagement with information, recommendations, and advice. Our findings about factors impacting successful alert design have utility to inform the development of local early warning systems.

**Supplementary Information:**

The online version contains supplementary material available at 10.1186/s12954-023-00761-6.

## Background

Drug prohibition creates illegal markets where it is impossible to control and regulate the quality and composition of products manufactured and sold [1]. Structurally and pharmacologically diverse new psychoactive substances (NPS) frequently emerge onto global markets, often with higher potency and more unpredictable effects than traditional products [2–4]. Unpredictable purity of traditional supplies, potential for cross-contamination during illegal manufacturing processes, and the proliferation of NPS and other compounds as cutting agents, adulterants, or substitutes pose new challenges for conventional drug market surveillance, regulatory controls, health, law enforcement, and community responses [2, 3]. While drug-related morbidity and mortality is often a complex interplay of contributing biological, psychosocial, and structural determinants of health and wellbeing, the risk of experiencing unwanted side effects and unexpected adverse events (e.g. toxicity/overdose) increases substantially when high-strength or novel compounds are consumed unintentionally [5–7].

Diversified drug production and increased market variability have been linked with drug-related morbidity and mortality worldwide [2, 8, 9]. For example, increased manufacturing of high-purity methylenedioxymethamphetamine (MDMA) is a likely factor contributing to reports of steadily rising MDMA/ecstasy-related mortality across the UK [10–12], while escalating drug-related mortality in Scotland has been driven by the increased availability of illegally manufactured ‘street’ benzodiazepine products [10, 13]. The most recent waves of North America’s ‘opioid overdose crisis’ are largely attributed to the contamination of unregulated drug supplies with novel synthetic opioids (e.g. fentanyl and related analogues) and (more recently in Canada) novel benzodiazepines [14–16]; prompting urgent coordination of innovative market surveillance and other public health policy and harm reduction responses [17–19].

Australia has also observed harms associated with the dynamic nature of unregulated drug market supplies [20–24]. For example, national coronial data have confirmed fluctuating MDMA-related mortality consistent with global indicators for MDMA/ecstasy market availability and increased purity [25]. Notably, in the summer of 2018–2019, MDMA-related toxicity (blood concentrations consistent with high-dose MDMA exposure) was the primary contributing factor to an unprecedented surge in hospitalisations and deaths of festival attendees in New South Wales, even when multiple drugs were present [26]. Although intentions about dose and polysubstance use were not recorded, high-dose MDMA/ecstasy consumption (e.g. ‘double-dropping’ multiple doses at a time), and ‘preloading’ substances before attending events is reportedly common in Australia among festival attendees [27, 28]. While these practices may reflect desires to enhance drug effects in the local context of historically low-purity MDMA/ecstasy supplies, they have also been reported as deliberate responses to punitive policing of drug use and possession at festivals, which can increase the risk of experiencing harm when the quality/composition of products is unknown [27–30].

Between 2018 and 2020, Victoria observed fourfold spikes in both MDMA- and NPS-related overdose deaths [31]. Nationally, the most common cause of NPS deaths is accidental toxicity involving novel cathinones (stimulants), phenethylamines (psychedelics/empathogens), and more recently, novel benzodiazepines—usually in combination with other substances [23, 24]. Since 2020, the Victorian Coroner has linked multiple deaths with unintentional consumption of NPS mistaken for other products purchased on illegal markets [32–35]. For example, an inquest held in 2021 confirmed the leading cause of death for five Victorians over 6 months (2016–2017) was unintentional consumption of a particularly dangerous combination of 25C-NBOMe (a phenethylamine) and 4-Fluoroamphetamine (4-FA: a cathinone)—reportedly mistaken for MDMA and/or psilocybin [32, 36]. At the time, local surveillance systems were not equipped to rapidly confirm the type and composition of substances contributing to earlier deaths before the same unique combination was linked with a cluster of hospital presentations for suspected MDMA overdoses in a single weekend in Melbourne in January 2017, and a sixth death 3 months later [36, 37]. Later, in 2020 and 2021, the same combination was identified in two other jurisdictions, prompting government warnings about ‘misrepresented’ MDMA supplies [38, 39].

Further market disruptions (e.g. adulteration/substitution) were anticipated during the COVID-19 pandemic, with restrictions expected to interrupt global production and supply chains—particularly in Australia, where illegal markets rely heavily on importation [40, 41]. While some Australians who regularly use drugs have reportedly observed changes in the price, availability, and perceived purity of some products [42, 43], the full impact of the pandemic on local markets is not entirely clear. Australia has several established drug monitoring systems primarily informed by research programmes, health and coronial data, wastewater analyses, and forensic seizures, but no single data set provides comprehensive and timely market intelligence. Collation of aggregate data can be slow, limiting the capacity for prompt detection and reporting of emerging trends and outcomes, and a local need for more timely and systematic data triangulation has been recognised [44]. Large-scale, coordinated, multidisciplinary collaborations (and more innovative technologies for early detection, monitoring, evaluating, and responding to emerging market trends) have been increasingly recognised as a necessary public health response to the dynamic nature of unregulated drug markets [44–47]. Rapid detection and early reporting of unexpected deviations in dynamically changing markets can proactively inform policy, policing, health, prevention, education, and harm reduction responses [17, 19, 48, 49].

Coordinated early warning systems (EWS) in Europe [50, 51], USA [18], and Canada [19] adopt features similar to public health surveillance and reporting of communicable diseases, with the capacity for prompt risk assessment and translation of emerging threats into tactical responses at local, national, and international levels [17, 19, 44]. EWS that operate within multiagency, cross-jurisdictional networks can publish timely communications (e.g. public alerts, warnings, notices, or advisories) to help mobilise individuals, communities, services, and governments into preventative or responsive action to encourage actions that can reduce the impact and/or incidence of adverse events [17, 19, 51]. Communications that offer specific, actionable descriptions and insights about known substances of concern (and/or reported harms) can facilitate rapid dissemination of relevant education, prevention, and advice to promote awareness of perceived threats and widespread information exchange to help reduce the impact of adverse events [17, 49]. However, in the current context of prohibition, criminalisation, and stigmatisation of people who use drugs, widescale public health initiatives have historically been overshadowed by the prioritisation of law enforcement approaches, resulting in reactive rather than preventative resource allocation for proactive health and harm reduction interventions [1, 52].

At the time of writing, no nationally coordinated EWS operates in Australia despite repeated calls for governments to support and fund coordinated early detection and reporting networks to prevent avoidable harm [32, 34, 35, 53–55]. Efforts to establish national partnerships for more collaborative surveillance and reporting have commenced [56, 57]. However, the coordination of data across Australia’s federated system is challenged by time delays and inconsistent reporting across jurisdictions and requires significant funding and resources to operate successfully [44]. Some states have piloted or currently manage local EWS—several of which have recently issued public health drug warnings and advisories—but these function in varied stages of maturity and capacity for ongoing, systematic data collection, analysis, and widespread, timely reporting [49, 58–61]. In Victoria, the Department of Health issued its first public drug alert in 2020 based on findings from a research project piloting a mobile testing laboratory at large-scale events as part of an emerging local EWS [62, 63]. Analysis of discarded ‘ground finds’ (e.g. samples not associated with identifiable individuals or forensic investigations) detected *N*-ethylpentylone—a potent cathinone known to cause harm when substituted for MDMA/ecstasy in Europe [64, 65] and New Zealand [66]—as the only psychoactive ingredient in distinctive green ‘UPS’ pills [62]. Alerts were issued onsite to event staff and patrons, and subsequently the general public. Since 2020, several public drug alerts have been issued in Victoria based on findings from the Emerging Drugs Network of Australia–Victoria (EDNAV), a multi-centre clinical registry established to improve surveillance and reporting of drug-related hospital presentations and their outcomes [59, 61, 67].

Effective and widespread communication is a key feature of successful public health program implementation [68], but evidence for best practice design and dissemination of drug-related risk communications (alerts) is limited [45]. Only a small body of research has qualitatively evaluated the effectiveness of drug alert messaging, format, and communications from the perspectives of people who use drugs, mostly in the context of alerts targeting people at risk of experiencing opioid overdose in North America [69–72]. This research has begun to demonstrate a need for carefully prioritising the values, interests, needs, and concerns of target audiences to maximise the reach of risk information and engagement with advice [48, 69–72]. Importantly, alert information or harm reduction advice that is perceived to be dated, inaccurate, unrelatable, irrelevant, or impractical can undermine trust and engagement with drug alert systems and information providers [70–72].

Health and community services may be ideal locations for opportunistic risk prevention and harm reduction conversations between practitioners and clients/patients who use their services, but providers may not be perceived as reliable sources of timely and reliable drug market information [69, 72, 73]. Best practice service provision within the alcohol and other drugs (AOD) sector is largely dependent upon workforce capacity for responding to emerging market trends, patterns of drug use, and changing community needs [74]. A key challenge for people working in AOD, health, and social work settings, however, is access to timely and reliable information about novel substances and emerging market trends [75–79]. For example, some health and community workers report feeling less confident responding to novel drug harms, particularly non-AOD specialists working in acute medical and mental health settings [78–81]. Alerts targeting these audiences may help to facilitate information exchange to improve workforce knowledge, confidence, and capacity for optimal planning, resource allocation, and provision of best practice care [17, 49, 77, 82]. There is, however, limited evidence for what tools and mechanisms might be useful for informing health and community workers about emerging trends to inform their work.

Services often rely on researchers to identify factors for successful implementation and evaluation of the interventions they deliver, but without consultation and adequate planning, researchers may be oblivious to important nuances and practicalities of the systems and individuals affected [83]. Consultative, participatory design and formative evaluation techniques can help to identify factors that may impact successful implementation of public health interventions [83–85]. This pre-implementation study therefore engaged health and community service workers in the co-design and testing of drug alert prototypes specifically for the translation of information about dynamic market changes and reported harms into practice to inform their work. This was the first phase of a larger pilot study (Rapid Drug Alerts for Victoria) for eventual design, implementation, and evaluation of alerts for an emerging EWS in Victoria informed by existing, available local drug surveillance systems. We present this paper as the first in a two-part series alongside Volpe et al. which explores key tensions that arose during our co-design process [86]. We recommend considering both papers for a comprehensive outlook on our experiences designing and evaluating drug alert prototypes in this context.

## Methods

### Aims

This formative evaluation aimed to tailor drug alerts specifically to the needs of people working within AOD and emergency medicine (‘urgent care’) settings. Specifically, it aimed to establish appropriate design, content, messaging, format, and dissemination mechanisms to support workers’ responses to drug-related harm in clinical and community settings, and identify factors that impact the successful implementation of a local alert system to improve health and community service workers’ capacity for responding to market changes involving high-strength, novel, or adulterated substances of concern.

### Co-design and evaluation frameworks

**Fig. 1:**
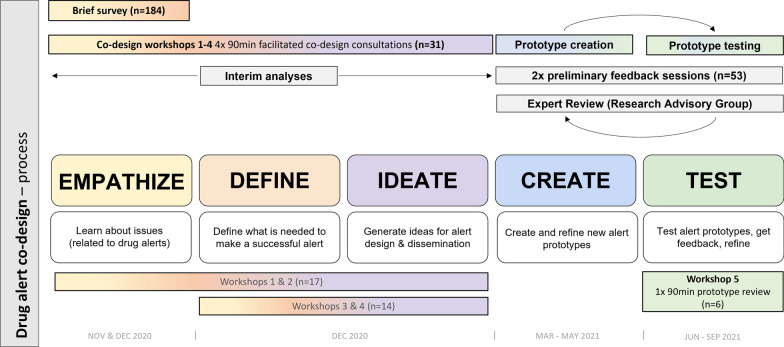
The drug alert co-design process. All co-design workshop participants (*n* = 31) were recruited from the initial brief survey (*n* = 184). Two preliminary feedback sessions were attended by participants attending an AOD service provider’s conference (*n* = 37) and a subset of the co-design workshop participants (*n* = 16). Six co-design participants then returned to attend the final ‘prototype review’ consultation (*n* = 6)

An iterative participatory co-design, parallel mixed-methods approach informed the development and testing of drug alert prototypes and helped to identify factors for successful alert implementation. Participatory co-design prioritises collaboration with target audiences to design successful products, systems, and services [85, 87]. A brief quantitative ‘needs analysis’ survey informed five qualitative co-design workshop consultations. Figure 1 illustrates five phases of our co-design process (empathise, define, ideate, create, and test).

Elements of the Consolidated Framework for Implementation Research (CFIR) were used to contextualise co-design findings and report key factors impacting the implementation of alerts targeting health and community workers [48, 88]. The CFIR is not commonly used in pre-implementation research but was used recently to inform drug-checking service design in Canada and the USA [89, 90]. It comprises 39 constructs across five domains that interact to influence program implementation: the intervention (in this case, drug alerts); the ‘outer setting’ (external and contextual factors); the ‘inner setting’ (structure and climate of the implementing organisation); individuals receiving and delivering the intervention; and the implementation process (e.g. planning, engagement, execution).

### Data collection, prototype creation and testing

Data were collected between November 2020 and September 2021. First, a rapid literature review informed the development of a short online survey (hosted on the Qualtrics® Insight Platform) to establish the relevance of alerts for health and community service workers and understand the scope of their information needs and preferences. Victorian alcohol and other drug (AOD) and urgent care (UC) workers were recruited via convenience and referral sampling methods. Four virtual co-design workshops were held in December 2020. We facilitated semi-structured workshop consultations using open-ended questions, opinion polls, and other interactive, ideas-generating techniques such as ‘brainwriting’—a timed activity where participants rapidly record independent responses to prompts before sharing them with the group [91]. Interactive software allowed participants to view and discuss responses in real-time (e.g. Mentimeter™, Google Slides, and chat functions). Workshops were hosted and recorded using Zoom video conferencing, and all media were transcribed verbatim. Interim analyses and researcher observations are fed-forward to inform future workshop discussions and alert prototype designs. We drafted alert prototypes based on co-design consultations and sought preliminary feedback during feedback sessions held between May and August 2021. Data from the interactive feedback sessions were reviewed by the research team and conflicting perspectives were escalated for discussion with an advisory group of experts working across government, health, AOD treatment, harm reduction, and research sectors. Unresolved tensions, alert dissemination and design were discussed with participants of the final ‘prototype review’ workshop held in September 2021. Unresolved tensions are presented in our companion paper, Volpe et al. [86].

#### Participants

Two core groups participated in the co-design process: (1) Survey respondents (*n* = 184); and (2) workshop participants (*n* = 31). Survey respondents were invited to opt into co-design workshop consultations and to receive alerts during subsequent implementation and evaluation phases of the larger pilot study described above. Eligibility to participate was defined by participants’ role as practitioners or managers in AOD or UC settings.^1^ Table 1 displays the characteristics of the survey cohort. Workshop participants were employed across AOD (77%, *n* = 24), UC (7%, *n* = 2), or both (16%, *n* = 5)^2^ settings in major metropolitan cities (Melbourne: 61% and Geelong: 10%, *n* = 22) and regional Victoria (29%, *n* = 9). Many participants held multiple roles across various organisations and settings, but most workshop participants identified as practitioners working exclusively in AOD settings or working in AOD roles within UC settings (65%, *n* = 20). Only two managers worked exclusively within UC settings.

**Table 1:** Survey respondents’ employment type (role) and workplace location by employment setting (sector)

Worker/workplace characteristics	Employment setting^a^			Total *N* (%)
	AOD^b^ only *n* (%)	UC^c^ only *n* (%)	Both AOD & UC *n* (%)	
*Employment type (role)*				
Practitioners (only)	128 (36)	12 (7)	4 (2.2)	144 (78)
Managers (only)	31 (17)	3 (1.5)	0 (0)	34 (18)
Both practitioner and manager	4 (2)	1 (0.5)	1 (0.5)	6 (3)
Total *N* (%)	163 (88)	16 (9)	5 (2.7)	184 (100)
*Workplace location*				
Major cities (Melbourne and Geelong)	118 (64)	14 (8)	4 (2.2)	136 (74)
Regional Victoria	45 (24)	2 (1)	1 (0.5)	48 (26)
Total *N* (%)	163 (88)	16 (9)	5 (2.7)	184 (100)

^a^Frequencies reported as a proportion of the whole survey sample (*N* = 184). ^b^AOD: alcohol and other drugs setting

^c^UC: urgent care setting

A third group provided preliminary feedback on alert prototypes at two feedback sessions held during the ‘create’ and ‘test’ phases of the project, comprising i) stakeholders attending a presentation at an AOD service providers’ conference (*n* = 37), and ii) a subset of co-design workshop participants who completed an optional self-paced, online feedback session (*n* = 16). Finally, six co-design workshop attendees returned for the final ‘prototype review’ consultation.

### Data analyses

Survey data were collated and cleaned using IBM SPSS 28 (*n* = 225). Cases were screened for contextual validity and consistency across responses, data completeness, and eligibility. Ineligible (*n* = 10) and blank (*n* = 31) responses were removed. Categorical data were presented as frequencies (%). Postcodes of workplace location were organised into discrete categories of ‘remoteness’ according to the Australian Bureau of Statistics’ Statistical Geographic Standard Remoteness Structure [92].

Workshop transcriptions, observations, and interactive data were analysed using SR NVivo 12. Based on an Iterative Categorisation approach [93], we used a deductive approach to develop an initial coding framework based on project research questions, workshop structure and further developed through inductive, open coding of the first workshop, fieldnotes, and observations. Refer to our companion paper for more on the coding framework [86]. Data were then coded independently but interpretations and conceptualisations were validated across researchers using triangulation methods [94]. Descriptive analysis involved grouping, regrouping, and reviewing points made in the workshops under each ‘code’ into logical thematic headings (objectives, context, audiences, alert features, content, messaging, design, format, and information dissemination). These ‘themes’ informed the basis for drafting and testing of alert prototypes. Key factors impacting successful alert implementation were then deductively coded to relevant CFIR constructs and domains to serve as a guide for informing future drug alert system design, implementation, and evaluation.

## Results

**Fig. 2:**
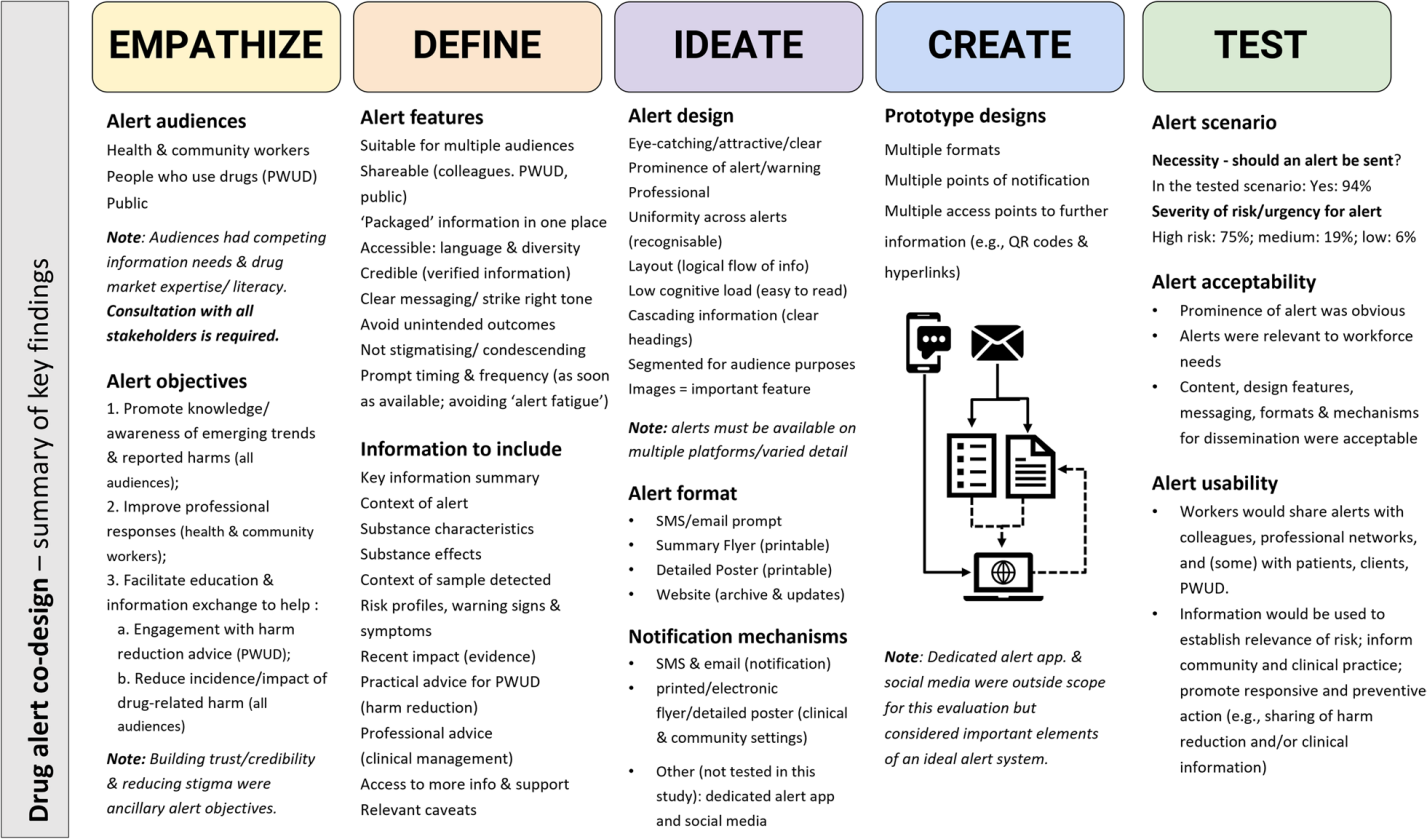
Summary of co-design findings

Key findings from the short survey, co-design workshops, preliminary feedback sessions and prototype review are summarised in Fig. 2. Rather than using CFIR terminology to describe determinants of successful alert implementation, key concepts (and themes) that emerged during each phase of the co-design process are presented in a cohesive narrative under the following headings:

- Establishing the need and context for drug alerts targeting health and community workers (alert objectives, context, and audiences)
- Tailoring alerts to health and community worker’ needs (alert features, content, messaging, and design)
- Notification mechanisms and timing of alerts (alert format and information dissemination)
- Developing and evaluating alert prototypes

Key findings (summarised in Fig. 2) mapped to each of the CFIR domains (and constructs) are presented in Table 2, ordered as they appear in the results narrative below: ‘Outer setting’ (needs and resources, access to information, contextual factors); Characteristics of individuals receiving and delivering alert information (knowledge and beliefs, attributes); Features of the alert (design, source, adaptability, relative advantage); Alert implementation (planning, engaging, executing, champions); and the ‘Inner setting’ of the service/system delivering alerts (e.g. structural characteristics, networks and communications).

**Table 2:** Guidance for future drug alert design (co-design findings mapped onto CFIR domains)

CFIR Domain (constructs)	Description	Co-design constructs	Key Findings
Outer setting (needs and resources, access to information, contextual factors) *CFIR constructs not examinable from study data, but recommended for future formative evaluations: Cosmopolitanism; Peer pressure; External policies & incentives*	Results from needs-analysis and key contextual factors that will influence successful implementation of alerts	Workforce needs Broader context for drug alerts Alert objectives	Drug market intelligence is important for informing clinical and community practice, but many health and community workers report limited access to timely drug market information (information currently accessed through secondary sources and often unreliable or unverifiable) Desire for receiving high-risk single-substance and drug trend alerts is strong Limited knowledge of emerging drug market information impacts credibility with clients/patients/consumers (AOD workers) and capacity for delivering effective clinical responses (UC practitioners). Alerts provide opportunity for building trust, credibility and rapport with people who use drugs and necessary information exchange Health and community workers have varied experience, knowledge and information needs regarding emerging drug market information (e.g. drug literacy; clinical management experience; and harm reduction knowledge) People who use drugs and public audiences have equally diverse substance-use experience, drug literacy/knowledge, and information needs Alert objectives should be clear and targeted: 1. Promote awareness of emerging market trends & reported harms (all audiences); 2. Improve clinical responses (health & community workers); 3. Information exchange & education (professionals, PWUD, public) to: a. Support informed decision-making (e.g. PWUD engagement with harm reduction/preventative action) b. Reduce incidence/impact of drug-related harm (professional & public) *NB: Alert objectives should also include building trust, credibility and engagement with harm reduction services and reducing stigma for people who use drugs*
Individuals receiving and delivering the intervention (knowledge and beliefs, attributes) *CFIR constructs not examinable from study data, but recommended for future formative evaluations: self-efficacy; individual stage of change; individual identification with organization*	Key characteristics of people receiving and delivering alert information (alert audiences) that will influence successful alert implementation	Alert audiences^a^ Beliefs and attitudes Knowledge and experience Other attributes	Audiences extend beyond the health and community services professional setting to people who use drugs and public audiences (friends, families, carers, etc.) Health and community practitioners are committed to reducing drug-related harms but responsibilities for preventing/reducing harm and perception of what this entails varies within and across sectors and roles (e.g. some UC workers prioritise clinical management of acute harm over providing harm reduction advice; some AOD workers are not harm reduction focused) Workers view themselves as ‘conduits’ for sharing information and education within and outside professional settings (colleagues, clients/patients/service users). They may access alert systems in multiple contexts Health and community practitioners are time-poor, often away from a desk for extended periods of time and can be hard to reach with a single platform/mode of communication
Intervention characteristics (design, source, adaptability, relative advantage) *CFIR constructs not examinable from study data, but recommended for future formative evaluations: Cost; Complexity; Trialability; Evidence strength and quality*	Key features of the drug alert that will influence successful implementation	Information source (credibility) Alert design: features, content, framing/messaging/tone, layout Relevance to stakeholders Adaptability Advantage of alerts	Information should be credible (evidence-based), realistic and relatable. The alert source must be trusted and have credible authority to issue alerts to facilitate engagement with recommended actions People who use drugs may be sceptical of healthcare providers with limited understanding of dynamic drug markets and distrusting of authorities that promote rhetoric about the ‘dangerousness of drugs’ in a prohibition world. Agendas must not appear alarmist Shareable outside a professional setting (e.g. ‘packaged alerts’ suitable for sharing with multiple audiences and settings who are not health and community workers) to minimise need for repurposing/translating/misinterpreting alert information Concise information but comprehensive inclusion of critical information/content (avoid withholding information or assuming hierarchy of knowledge among audiences) Language should be accessible to all audiences (consider communities with diverse literacy, physical and cognitive abilities, education, and cultural backgrounds) Messaging must be clear, engaging, and ‘strike the right tone’ (not sensationalised/hyperbolic, condescending, or stigmatising). Avoid unintended outcomes (e.g. stigmatising people who use drugs, sensationalising risk, promoting drug-seeking behaviour and/or unrealistic outcomes) Design should be attention-grabbing, professional, recognisable and ‘obviously an alert’. Uniformity of branding/design and prominent severity indicators promote alert identification and action. ‘Professional’ designs lend to credibility (but public audiences of people who use drugs may favour less-formal designs). Unnecessary logos and branding should be avoided Layout should flow logically to minimise cognitive load required when filtering through information (easy to read, clear actionable headings/ cascading information with key information at the top, information segmented for utility and relevance to multiple audiences) Tailored for multiple audiences (workers, people who use drugs, public). Each alert must be adapted to ensure contextually and situationally relevant content, messaging, recommendations and advice Alerts increase exposure to emerging drug market information and help to verify anecdotal reports about local drug markets and facilitate more efficient and reliable communication within and across health and community settings. Alerts are valuable tools for building trust and rapport with clients and patients; prompting conversations and sharing credible, evidence-based information to promote risk perception, encourage individual behaviour change/responsive action. More broadly, they may also help to reduce inadvertent stigma, and potentially irrelevant hyperbole about the ‘dangerousness of drugs’
Process of implementation (planning, engaging, executing, champions) *CFIR constructs not examinable from study data, but recommended for future formative evaluations: Executing; Leadership; Reflecting & evaluating*	Key features of alert dissemination that will influence successful implementation	Alert format/dissemination mechanisms Timing and frequency Consultation Trust in alert system Alert champions	Alerts must be disseminated on multiple platforms and in various formats (electronic and printable materials) Email, SMS, smart phone applications and social media are preferred tools for receiving alert notifications. Systems should consider technical limitations of each platform and consider broad communication strategies for providing alerts on platforms relevant to a range of different stakeholder groups (e.g. SMS has limited information; bulk emails with attachments can be blocked; paper & electronic formats are preferred for different scenarios) All alert formats should include access to more detailed, contextual information when available. A *closed information loop* should provide access to further information at all points where someone might engage with the alert Alerts must be timely and relevant (ideally issued within 2 weeks of an event happening), and alerts should be sent as soon as information becomes available. Processes should not delay publication and dissemination of time-critical emerging drug market information Prominent risk severity indicators (tiered notification systems) can highlight situational urgency, facilitate prompt responses and help to minimise ‘alert fatigue’ Websites (centrally managed, dynamic online alert repositories/archives) are critical adjunct to alert systems and should segment information into categories of relevance for different audiences so individuals can opt into the type and level of information required Trust in alert systems can be established with consistent and reliable branding, relevant and relatable messaging, evidence-based verifiable information, engagement of key stakeholders, and transparency about source of alert information The alert system should consult with key stakeholders (e.g. health and community service professionals, peer support organisations, and community) to build trust in the alert source Alert ‘champions’ may facilitate communication networks and more efficient information exchange
Inner setting (structural characteristics, networks and communications) *CFIR constructs not examinable from study data, but recommended for future formative evaluations: Culture; Implementation climate (e.g. leadership, available resources, readiness for implementation); Structural characteristics*	Key characteristics of the service/system delivering alerts that will influence successful implementation	Alert source (single, centrally managed structure) Communication networks	Information should be centrally managed and disseminated by a single information source to minimise the need for filtering through multiple resources/notifications Health and community service professional networks and organisations have varied information-sharing systems and procedures for communicating drug market intelligence (e.g. workplace communication systems between AOD services and local law enforcement or emergency services are highly variable). Alerts may help to support improved information sharing across and within networks Systems must consider processes to facilitate information flow within and across networks, organisations and community settings (beyond the alert system) Alert systems require networks of multiple information sources to triangulate and verify alert information

^a^Alert audience in this context were people who receive alerts and disseminate drug risk information (e.g. health and community workers, people who use drugs, and publics)

The CFIR domains are presented in the order they were reported in the results narrative. Not all CFIR constructs were examinable from the co-design study data. The exhaustive list of CFIR domains and constructs are outlined in Damschroder et al. [88]

### Establishing the need and context for drug alerts targeting health and community workers

First, we present quantitative data from the initial survey to establish this cohort’s perceived need for alerts targeting the health and community workforce (*n* = 184). Nearly all survey respondents agreed it was important for timely information about unexpected substances and emerging drug market trends to be available to workers (98%, *n* = 179) and managers (94%, *n* = 171). Almost all survey respondents reported that this information would be used to inform clinical responses (93%, *n* = 130) but many also reported inadequate access to timely market information about changing drug market characteristics and trends (64%, *n* = 117). Nearly all participants were interested in receiving notifications/alerts about emerging drug market trends and/or unexpected adulterants or high-strength/novel compounds in circulation (96%, *n* = 177) with most opting into receiving alerts during implementation and evaluation phases of the larger pilot study (76%, *n* = 140). Many had recently accessed information about drugs trends or seen alerts about suspected market adulterations, high-strength, or novel compounds, but information sources varied (see Table 3).

**Table 3:** Survey respondents’ reported source of recently accessed drug market information

Information source^a^	Drug market information	
	General drug trends data *n* (%)	Drug alerts/unexpected market changes *n* (%)
Clients/patients	103 (74)	37 (33)
Professional networks (conversations with colleagues/internal workplace communications)	102 (73)	72 (65)
AOD agencies (responsible for information dissemination)	84 (60)	63 (57)
Harm reduction/peer organisations	75 (54)	44 (40)
Drug institutes (research organisations)	56 (40)	21 (19)
Health/government agencies	38 (27)	36 (32)
Media	29 (21)	24 (22)
Other	10 (7)	6 (5)
Total *N*	139	111

^a^Participants could select multiple options

Data presented for participants who had recently access drug market information or seen high-risk single-substance alerts (in the last 12 months)

Early co-design workshop discussions revealed participants’ perspectives on the relevance of alerts for distribution to healthcare and community service workers. AOD practitioners and managers considered themselves conduits for sharing information with a range of professional and community audiences (e.g. colleagues, professional networks, patients, and clients). They considered alerts to be important tools for increasing exposure to information about emerging market trends and associated drug-related harms:

…an increase in trend[s of] people presenting in hospitals with a certain type of overdose or unusual reactions, […] they’re the things that we need to be alerted for. [UC manager, #42]

Three key alert objectives were identified by participants during early co-design consultations:

1. To promote awareness of market intelligence about high-strength, novel, or adulterated substances (perceived threats) and reported harms (adverse events) among health and community services, their clients, patients, and the general community;
2. To improve workforce responses to drug-related harm (e.g. ensuring optimal planning and resourcing for adequate service provision in clinical and non-clinical settings; refining clinical assessment and patient management pathways for specific drugs of concern in UC settings; scaling up responses to perceived or detected threats);
3. To facilitate information exchange and education (across *and* within clinical and community settings) about appropriate, evidence-based harm reduction advice and prevention interventions that can:
4. Support more informed decision-making about substance use among people who use drugs (e.g. encouraging community engagement with harm reduction behaviours and other preventive actions); and
5. Help to reduce the incidence/impact of drug-related harm in the community more broadly (e.g. overdose, hospitalisation, or death).

Improving clinical responses was particularly relevant to UC workers, who frequently discussed the opportunity alerts offered in terms of maximising patient and community safety:

I think that it would be good just to have […] paramedics and the other healthcare workers knowing what [emerging trends] are, what their reactions are. What the modalities to treat them are. Just so people can be prepared. […] it’s best practice for the patient if we know what we’re doing ahead of time. [UC manager, #42].

AOD and UC workers discussed how alerts could facilitate more efficient and reliable communication within and across healthcare and community workplace settings. Several practitioners —particularly those in regional settings— noted limited awareness of novel or high-strength drugs in circulation until after adverse events occurred locally. Challenges associated with verifying information about drug markets were discussed, with many workers noting they often rely on anecdotal reports from clients, patients, or colleagues, and must make ad hoc decisions about information reliability. In some settings, formal or informal workplace communications about market trends were common, but direct communication lines between AOD services and local law enforcement or emergency healthcare services varied. As one AOD practitioner noted:

…we have two sources. One from [accident and emergency departments] […] they can’t tell us what’s in the substance, all they can tell us is this is happening […]. The other one that we pass on is client information, of course around ODs but also around other substances that may or may not be what they’re supposed to be […] a lot of the information we get from clients that we pass on, sometimes we find is very valuable yet other times, it may not be as valuable […]. We think it’s better to tell clients if we do hear of particular substances being sold that aren’t good […] clients usually know about trends before we do, so yeah. [AOD practitioner, #65]

Importantly, some AOD workers—especially less experienced workers, or those not working in harm reduction roles—expressed concern that their limited knowledge of novel substances and emerging market trends and related harms impacted their credibility when discussing substance use with clients and patients who use drugs. Reliable and practical alert systems were considered valuable for building trust and rapport with clients and patients who may be sceptical about healthcare providers’ knowledge of dynamically changing drug markets. AOD practitioners and managers discussed the value of alerts as useful prompts for conversations about credible, evidence-based harm reduction information within clinical and community settings.

Participants also reflected on the value of alerts as useful prompts for sharing reliable and evidence-based information across and within their professional networks. Specifically, AOD and UC workers described alerts as tools that could provide time-poor workers with reliable resources to have ‘on hand’ to refer back to as needed. It was inferred that alerts may even help to reduce inadvertent stigma, and general hyperbole about the ‘dangerousness of drugs’ that can impact consumers’ engagement with harm reduction information and advice—especially risk information delivered by healthcare providers and other ‘authorities’.

### Alert audiences

A point that repeatedly emerged as important to participants was that alerts must be suitable for dissemination beyond the health or community service setting—they wanted alerts that were ready for immediate on-sharing directly with people who use drugs. AOD workers did not want to withhold critical information from audiences who may benefit:

a lot of the content that we’re talking about is aimed at us as a workforce, which I think is huge and it’s really important, but I don’t know why but naturally my mind continues to go back to the consumer and getting those alerts directly to the consumer. [AOD manager, #25]

For AOD practitioners especially, it was almost impossible to separate alerts for health and community services from public alerts that target people who use drugs in the community, and it was critical for alert information to be packaged in a way that could be shared directly with people who use drugs as soon as possible. For UC workers, ‘reducing harm’ generally meant more effective clinical responses to maximise patient safety, but their support for sharing alerts beyond the clinical setting was clear:

It would be really nice to get an alert sent to the community, to the people at the front face, rather than us having an alert as clinicians and managing the after-effects of what’s happened […] to reach the people that it absolutely affects [UC manager #47].

Participants recommended that drug alerts should be packaged into formats relevant for sharing directly with all audiences (communities of people who use drugs, their friends, families, carers, and the wider community). Time-poor practitioners were not interested in repurposing or translating alerts into shareable information, and were worried about the risk of diluting, omitting, or misinterpreting critical information. They wanted ‘packaged’ information to avoid reproducing information into appropriate formats for different audiences. One AOD manager recommended that alert systems ‘spoon feed people [and] make it easy for [us] to be able to share this information’ [AOD manager, #57].

### Tailoring alerts to health and community workers’ needs

Participants discussed at length their own information needs and the perceived needs of communities they work with regarding content to include, appropriate messaging, design features, and preferred formats for alert notification and information sharing (dissemination).

### Content

During the co-design workshop discussions, it became clear that alert systems should not assume a hierarchy of knowledge or information needs across or within relevant stakeholder groups. Participants were adamant that information must not be withheld from any potential audiences, and repeatedly prioritised the need for alerts that offer clear, contextual information relevant to all audience groups. Across several workshops, participants identified ‘critical information’ to include in all alerts to provide important context to promote engagement with alert information and advice. A comprehensive summary of the content that co-design workshops deemed necessary for inclusion is provided in Table 4.

**Table 4:** Critical information participants required for drug alerts (alert content)

Content elements	Information to include	Key findings
*Key contextual information about substance/s and harm/s*^a^		
Context of alert	Alert date Date of sample/case Location of sample/case Data/information source	Must not identify individual cases Data/information source is critical for establishing credibility of alert information and alert source and relevance
Substance characteristics	Substance class Common routes of administration, forms, dose Alternative drug names	
Substance effects	Common experiential effects (desired) Undesired experiential effects	Undesired effects generally considered important by all participants, while desired effects were not considered relevant among UC workers
Context of sample detected	Form (including images) Details of drug information and analysis ‘Sold as’ (if known) Fillers/adulterants (if known)	Images are important, wherever possible Caveats should be provided about the inevitable variability of substance form/appearance in unregulated drug markets
Risk profiles	Warning signs and symptoms of toxicity At-risk populations Experiential effects that might indicate danger Signs and symptoms of toxicity	
Recent impacts (evidence of harm)	Known harms (general) Quantifiable evidence of harm (e.g. hospitalisations, mortality) Other credible and relevant reports of harm	Must be relatable and realistic Must not identify individual cases Builds credibility of the alert
*Clinical management recommendations*		
	Characteristics of clinical presentations Potential drug interactions Best practice treatment pathways Complex or unique medical management strategies Post-acute care/discharge recommendations	Support assessment of presentations/ identification of differential diagnoses Experience from other clinical settings/ jurisdictions should be shared, where relevant
*Harm reduction advice*		
	Universal harm reduction advice Substance-specific harm reduction advice Recommended preventative actions How to recognise harm, respond and get help	Must be relevant to substance of concern Advice must be relevant, relatable, and achievable for lay people experiencing/witnessing adverse events
*Access to further information and services*		
	Substance-specific information (all audiences) AOD support helplines (people who use drugs) Clinical advisory services (practitioners) Archive of alert history and related information Links to online information	Context about services offered should be provided Services listed must be aware of alerts to ensure readiness to respond QR codes or hyperlinks are useful

^a^All the information included under each subheading may not be available in all situations, or it may be inappropriate to publish information that can identify individual cases (particularly in regional settings), but all categories and segments of information should be included in detail, where possible

Information included will vary on a case-by-case basis, and the level of detail to include will depend on the alert format (e.g. SMS prompt should include highest-level contextual information, substance class, and access to further information via direct links; the summary flyer should include simple, actionable headings, with some context—substance information, recent impacts, etc.—and basic harm reduction advice with access to further information (QR codes/links); while the detailed poster should include all information available with QR codes and links to website

Based on rich discussions about what must be included to satisfy audience needs, we segmented alert information under four headings based on utility and purpose, rather than separate alerts for different audience groups. Specifically, we developed four categories of information for inclusion: (1) *Key contextual information about identified substance/s;* (2) *Clinical management recommendations*; (3) *Practical harm reduction advice*; and (4) *Further information and support*.

*‘Key contextual information about identified substance/s’:* Co-design workshop participants identified ‘must have’ content critical for providing situational context about identified substances and the context of the alert itself (refer to Table 4 for key findings about alert content). Including this high-level detail was deemed to build trust in the credibility of information provided. Workshop participants noted that transparency about the source of information (e.g. police, hospital, government) and quantifiable evidence for impact and local harms (e.g. adverse events, hospitalisations, or deaths) were important for promoting the perception of relatable risk/threat and engagement with recommendations/advice.

One notable feature that was repeatedly discussed was the inclusion of images of tested samples (where available) to support the identification of potentially harmful substances in the community. Experienced harm reduction workers also noted the importance of including caveats about the inevitable variation in drug potency and form in unregulated markets alongside image descriptions. It became apparent to us during prototype design that some ‘critical information’ may not always be available in all situations, and we note that information included will vary on a case-by-case basis. Members of the research advisory group also noted that care should be taken to avoid the inclusion of information that could identify individual cases/events particularly in regional setting (e.g. noting specifics about hospital presentations).

*‘Clinical management recommendations’* were not only relevant to practitioners and managers working in clinical settings. It was noted that this information was useful for promoting health literacy within the community and ensuring accessibility of information for all audiences. One UC manager noted ‘I think the [clinical] information should be available to consumers and […] they can use it if they want to use it. I don’t think clinicians should have any more information than consumers in regards to information around choices that they may make.’ [UC manager, #42].

*‘Harm reduction advice’* was also considered by workshop participants to be universally relevant to all professional and community audiences, regardless of their professional role or experience. One practitioner noted that ‘making it as easy as possible to have those [harm reduction] conversations […] means it’s probably more likely to happen’ [AOD practitioner, #61]. A key concern was that harm reduction messaging must not be ‘too generic’ or too focused on severe but unlikely consequences (i.e. mortality). Discussions about the inclusion of harm reduction advice that offers practical and achievable solutions should be specific to the substance of concern, and should address the needs and preferences of communities at greatest risk of experiencing harm, to support the prevention of adverse events in the first place:

…we’re not actually saying what can you actually do to be safe in the instance that these substances are out there […]. [Some alerts focus on] overdose as a major risk but not necessarily other types of harm reduction advice - how to look after your friends and changing different habits to kind of avoid overdose in the first place. [AOD practitioner, #61]

*‘Further information and support’:* Practitioners and managers noted the importance of avoiding assumptions about audience expertise or information needs and empowering audiences to seek out more information if desired. Links to further information and support services were important inclusions but deemed most useful if context about the relevance of the support service is provided. Several AOD workers noted that any services listed should be adequately briefed about emerging concerns so they are equipped to respond to alert-related enquiries.

### Message framing and narrative

Throughout the workshops, participants expressed desires for alerts with consistent messaging that strikes a balance between a sufficient focus on relatable evidence of harm, while avoiding sensationalising serious but unlikely harms and scaremongering hyperbole about the ‘dangerousness of drugs’ or promoting unlikely consequences of use and/or recommending unachievable outcomes/actions that could jeopardise engagement with alerts and credibility of the information provider. In summary, participants wanted messaging that provides practicable, relatable, and relevant action-based directives, rather than tokenistic or generic advice. It was important to practitioners and managers that language should be clear and not overly medicalised, using simple narratives that are easy to follow and do not require complex interpretation.

### Design considerations

In early co-design sessions, participants reviewed the positive and negative features of previously issued alerts to inform design elements for the current study. In summary, participants wanted immediately recognisable, ‘attention-grabbing’ alerts, with uniformity of design and ‘professional’ branding. Participants noted that designs should be equally engaging for audiences beyond healthcare and community service settings, who may have different alert design preferences. Workers preferred clear headings and a logical flow of information that directed the eye to relevant content. ‘Executive summaries’ and actionable headings were considered important to help workers scan for the relevance of information and quickly decide about resulting actions (e.g. accessing more information, or information-sharing with others).

We established from discussions that the layout should flow logically, with effective use of white space and actionable headings to draw the eye to important information and reduce cognitive load. ‘Cascading information’ (core information at the top) segmented under headings helped participants navigate directly to the information most relevant to them. Unnecessary logos and excessive branding were thought to overpower important messages, and participants noted these should be avoided. Discussions repeatedly raised the importance of accessibility considerations to ensure inclusivity for communities with diverse literacy, physical and cognitive abilities, education, and cultural backgrounds.

Although the focus of this study was alerts for health and community service workers, workshop participants strongly and repeatedly asserted the importance of engaging people with lived/living experience in the design and dissemination of drug alerts. At all co-design workshops, discussions included the importance of including perspectives from people with lived- and living-experience of drug use to ensure messaging and design were relevant to them. As one AOD practitioner noted, ‘[peer input] will actually help you to analyse the message you’re doing.’ [AOD practitioner, #112].

### Notification mechanisms (alert format and dissemination)

Prior to co-design workshops, survey respondents had identified preferred formats for alert notifications that informed what notification mechanisms were discussed at the workshops. Email was overwhelmingly the most popular mechanism identified for information dissemination (90%, *n* = 159), followed by dedicated smartphone ‘drug alert’ applications (48%, *n* = 85); short text messaging service (SMS: 40%, *n* = 71), internal workplace communications (31%, *n* = 55), and social media (24%, *n* = 42). Developing a dedicated drug alert application and testing widespread social media communications were beyond the scope and capacity of the current study, but their value was discussed at the co-design workshops, and participants noted that having a website repository of historical alerts was a critical feature for future alert systems design.

Co-design workshop participants weighed up the benefits and limitations of different platforms for receiving alert notifications. Of the formats feasible for development during the current study, SMS and email were considered the most practical methods for receiving alert notifications about emerging market trends, and unexpected substances in circulation. It was noted that alerts issued from a single, reliable information source can be flagged as priority reading by time-poor professionals, who can easily recognise the source of information and use these methods as prompts to engage with other formats for more information:

if I knew that the source was coming from drugalerts.com I’d look at it, because it’s relevant. [AOD practitioner, #112].

The value of SMS in addition to email notifications was highlighted. Participants noted a brief text message to their mobile phone can reach workers almost immediately, regardless of their location (i.e. when away from their desk). However, SMS are generally limited to 160 characters. Participants discussed the utility of having a text message that can easily be on-shared even though the information included would be limited. Notably, SMS prompts were described by participants as useful for prompting engagement with other alert formats (e.g. email, flyers, websites), and forwarding information to clients/patients—especially those who are not actively engaged with services or have limited access to email/Internet:

a sentence or two that could be copy/pasted into a text message would be really useful …not everyone has a smartphone [to] click a PDF [electronic alert poster] and follow through the links... [AOD practitioner, #46]

While emails provide more comprehensive information and context than text messages, AOD and UC workers noted that their work regularly takes them away from the desk for extended periods, and returning to an inundated inbox can be overwhelming and important information can be missed if the title is not clear and recognisable. Several participants noted a barrier to email notifications: large organisations often block bulk emails sent with attachments. Including relevant summaries in-text with hyperlinks to access electronic/printable document formats (PDFs) may be preferred over attaching documents to alert notifications.

We’re just constantly bombarded with emails, and if it was an alert [only] by email it would definitely get missed. I think I’d really struggle with that, but if it was that SMS or app based, then that’s going to be something that I’ve got access to, like I’m on the road all the time but I’ve got access to my phone and I can see that very quickly and then recognise whether I need to then forward that on or pass that information onto the clients. [Regional AOD practitioner, #159]

Regardless of preferred notification mechanisms, workers wanted alerts to be available on multiple platforms and in various formats for different purposes and audience groups:

We’ve got different target audiences that we deal with and so I can see that SMS is perfect for some […] but in my workspace, I would like the PDF [detailed alert]. I’d like a detailed one for my benefit but then a summary one that I can then distribute to [emergency first responders] that work with me […] and if it’s relevant, send that information on to our medical director to say, here you go, do we want to put this out to [the entire organisation]. [UC manager #42]

### Print versus online materials

Despite the obvious benefits of digital communications for rapid notification and easy dissemination, printed materials (e.g. posters/flyers) were important for sharing information within health services and distributing information more broadly with patients or clients. For example, participants noted that printed materials can be displayed offline in a range of clinical and community settings (e.g. public notice boards); used as prompts in face-to-face conversations; and provide a quick reference point to refer back to when needed. For one participant printed information was ‘important [so] that we can give it out to clients that we are unable to give digital things to’ [AOD practitioner #7]. But on the other hand, printed materials may not be useful to others:

I’d be more likely to forward an alert via SMS to a client I was worried about than I would be to hand them a bit of paper, probably […] I can send that alert to them and I can follow it through with a phone call [...] a higher percentage of clients I work with don’t have great literacy, so written information for them is not going [to be ideal] [AOD practitioner, #22]

Participants did not consider websites particularly useful mechanisms for alert notifications, but it became clear that a centrally managed, online repository of alerts is a necessary requirement for accessing alert archives ‘rather than having to go back through an unwieldy email inbox’ [AOD manager, #61]. Participants noted that websites were ideal for hosting the highest level of information, and pages could be segmented into categories of relevance for different audiences so individuals can opt-in to the type and level of information they require:

in my mind I imagine an email that says dangerous drug alert […] and then it prompts people to go to the website where there’s more information. [AOD manager, #57]

…once they click onto the website there’ll be specific information for consumers, specific information for health professionals and they can get more information if they want. [AOD practitioner, #95].

### Timing of alerts

During the initial brief survey, respondents were asked about appropriate time delays when issuing alerts. Most survey respondents (89%, *n* = 159) considered 2 weeks or less an acceptable delay for alerts about high-strength, novel, or adulterated substances in circulation, and many wanted alerts issued as soon as the information was available (77%, *n* = 137). While workshop participants confirmed a need for immediate information, it was noted that processes to publish alerts in multiple formats should not delay rapid dissemination of time-critical information. Participants also raised concerns that alerts issued too frequently could dilute ‘urgency’ of important information and lead to disengagement from alert systems altogether. We noted an important tension between ‘gatekeeping’ important information while maintaining audience engagement and minimising ‘alert fatigue’ (disengagement due to information overload or too many alerts sent too frequently).

### Developing and evaluating alert prototypes

Based on the co-design workshop discussions, we drafted three initial prototypes for a hypothetical alert scenario derived from an actual alert issued in another jurisdiction to ensure the authenticity of the information presented [95]. Prototypes for an SMS prompt and email notification were developed. These provided summaries of alert information to elicit engagement with more detailed alert information (presented as an electronic PDF/poster that could be downloaded and printed).

### Preliminary feedback

The SMS/email prompts and detailed alert poster were presented to stakeholders attending the two preliminary feedback sessions who participated in polls and discussions about the utility and design of each of the drafted alert prototypes (*n* = 53). Feedback provided implied that the email/SMS prompts were easily identifiable as alert information to elicit further action. When polled, the majority of stakeholders agreed that they would likely forward these to colleagues (SMS: 88%; email: 92%), or share/discuss them with clients/patients (SMS: 75%; email: 80%). Responses about the detailed alert were, however, mixed. When asked to describe the poster/PDF in a few words, some stakeholders reported it was ‘important’, ‘informative’, ‘clear’ and ‘eye-catching’, while many considered it ‘busy’, ‘text-heavy’, ‘dense’, or ‘too much’. Others offered feedback that the ‘severity’ of alert (i.e. perceived risk/threat) was not immediately obvious.

Based on the feedback received, we modified the alert prototypes. To resolve potential for ‘information overload’–and possible disengagement from alert information—we developed a ‘summary flyer’ with more ‘white space’ that offered a more concise summary of alert information with actionable, directive headings for more widespread dissemination beyond the clinical/community service workforce. Secondly, to facilitate more rapid assessment of alert ‘severity’ and level of ‘urgency’, we implemented a colour-coded ‘severity indicator’, similar to those used in Australian bushfire alert communications [96]. We considered what information should be included in each alert format to effectively convey context for the alert, relevant information and advice. In summary, based on information gathered from earlier co-design discussions, we concluded that SMS prompts should include the most concise detail to provide urgency and context of the alert and offer links to access more information; the summary flyer should include simple, actionable headings, and basic context to encourage further information-seeking (if desired) and engagement with basic clinical management or harm reduction advice; while the detailed poster/PDF should include all available information.

### The alert ‘journey’ (accessing alerts and sharing information)

**Fig. 3:**
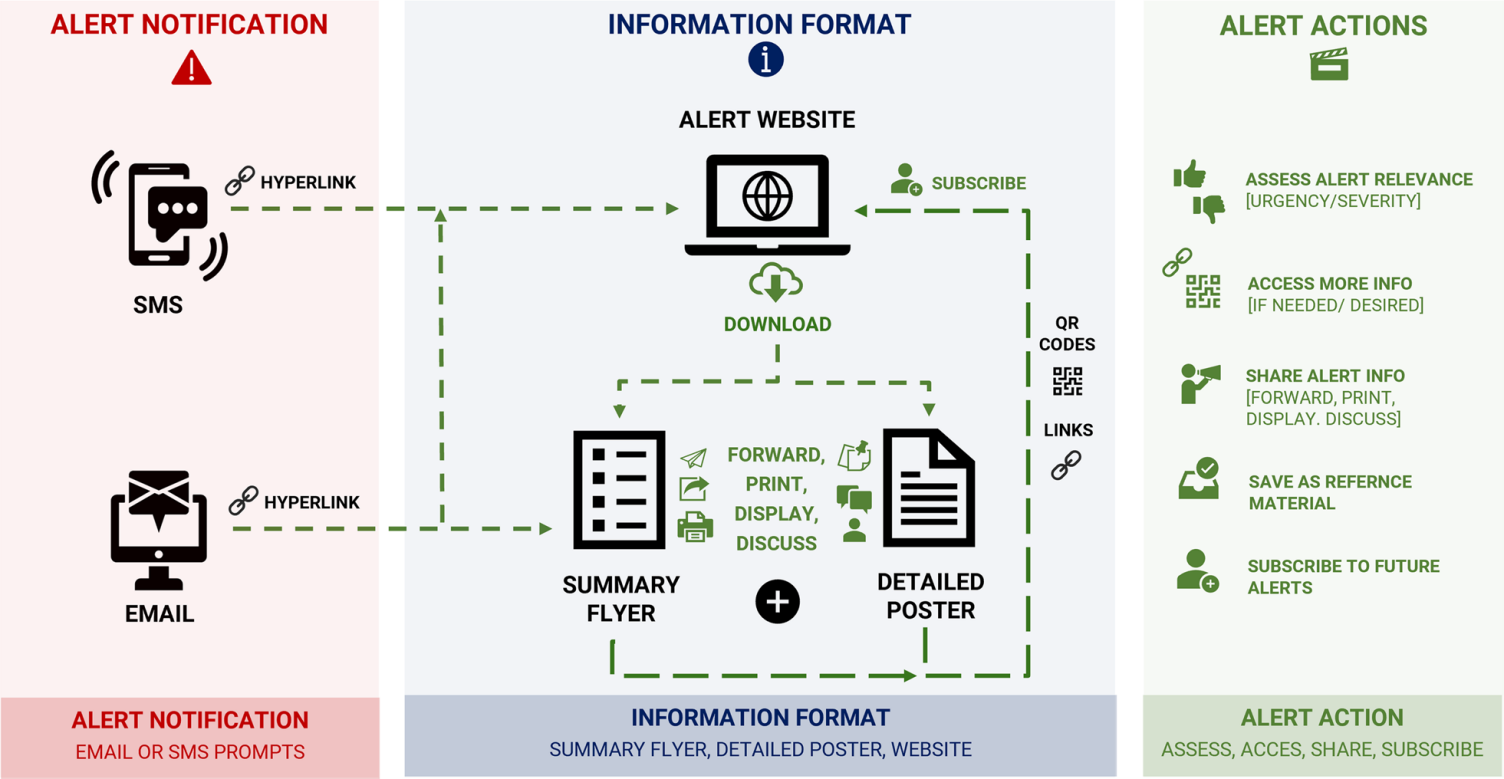
The ‘alert journey’: Accessing alerts in various formats and resulting action after engaging with alert information. There are a few scenarios in which audiences might commence engagement with the alert system. Direct hyperlinks and/or QR codes facilitate low-barrier access to all levels of information, and opportunity for further action, regardless of the format in which a person first engages with an alert information: (1) receiving alert notifications (email or SMS prompts); (2) observing alert information in various formats in clinical or community settings (summary flyer, detailed poster, alert website); and (3) actions taken after engaging with the alert: evaluating relevance; accessing more information; sharing information with others (forward, print, display, discuss); or subscribing to future alerts

When drafting alert prototypes, we realised that audiences may commence engagement with alerts at any point from when an alert is first issued. For example, subscribers may receive electronic notifications via SMS or email prompts, while others may observe alerts for the first time in workplace or community settings (e.g. summary flyers, detailed alert posters, or alert websites). Given previous assertions from co-design participants that alert information should be accessible to all recipients, we felt it was important to facilitate low-barrier access to all levels of information, regardless of the alert format people first engaged with (SMS/email prompt, summary flyer, detailed poster, or alert website). Active hyperlinks were included in all electronic materials, and ‘quick response’ (QR) codes (scannable barcodes to navigate directly to online resources) were included on the summary flyer and detailed poster so that static (printed) materials could be scanned using a mobile phone for immediate access to more information [97]. Figure 3 illustrates the different stages where audiences may first engage with alert information, and outlines possible actions taken when first receiving an alert: (1) receiving alert notifications (SMS/email prompts); (2) observing alert information ‘in the field’ (e.g. summary flyers, detailed alerts, or alert websites); and (3) actions taken after first encountering with the alert (e.g. evaluating relevance, accessing more information, sharing information with others, or subscribing to future alerts).

### Prototype testing

**Fig. 4:**
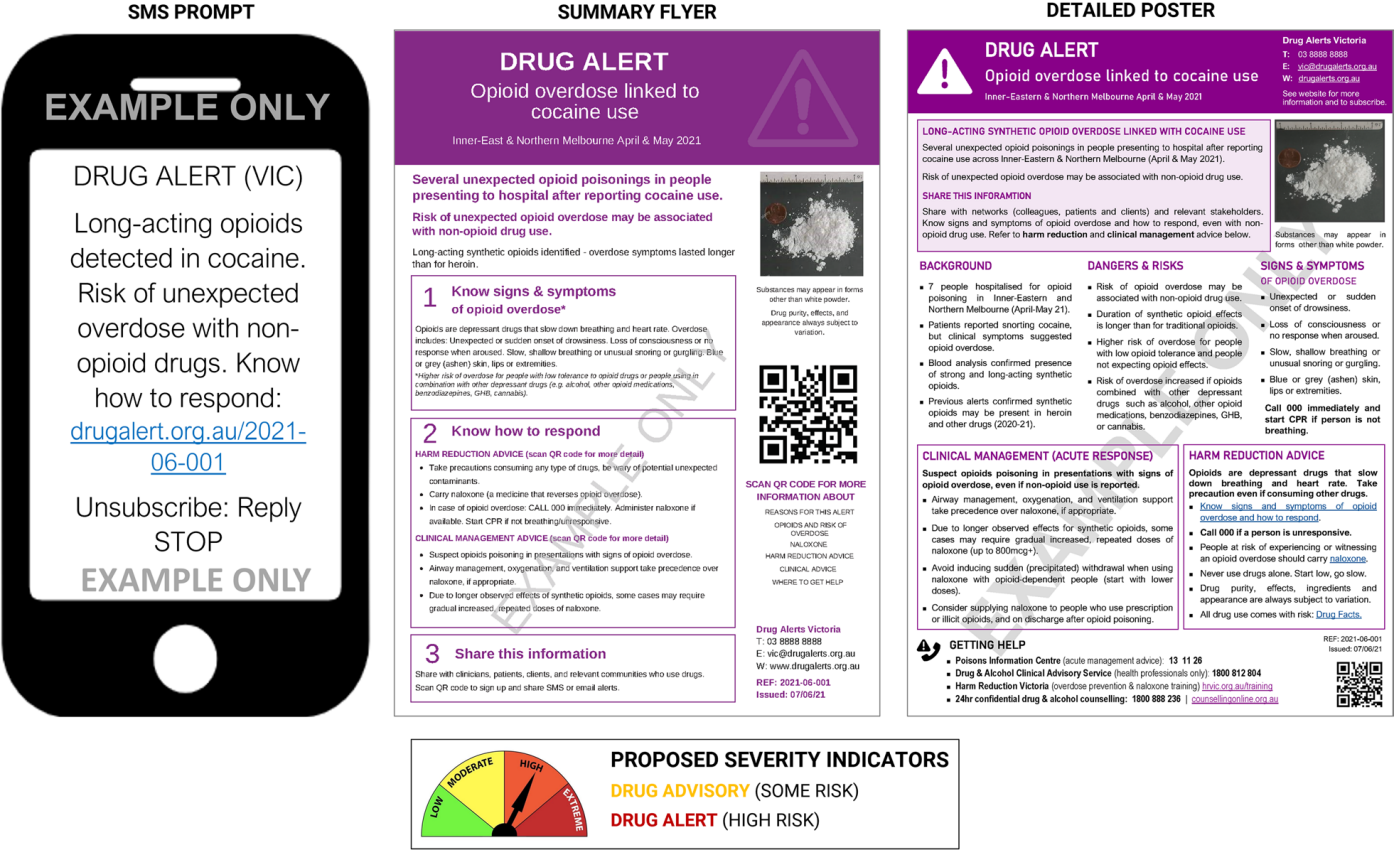
Final alert prototypes with proposed severity indicators (SMS prompt, summary flyer, and detailed poster)^a^. ^a^Full size A4 versions of flyer and poster are included in supplementary materials online (Additional files 1, 2)

Three alert prototypes with varied levels of information were presented for testing at the final ‘prototype review workshop’: (1) SMS Notification; (2) Summary Flyer; and (3) Detailed Poster. As emails ended up being reproductions of content included in the detailed poster, they were not presented for testing at the prototype review. The three final alert prototypes are displayed in Fig. 4, and larger versions of the summary flyer and detailed poster are provided as supplementary materials (Additional files 1, 2).

Conflicting perspectives about the inclusion of ‘critical content’ while avoiding ‘information overload’ and disengagement from alert information were discussed with participants who agreed the desire for succinct and concise summary information should not come at the cost of withholding important information. Participants echoed comments from earlier co-design consultations: the level of information included in the detailed alert was necessary for providing important contextual information and credibility of alert information for use in a service provision context; however, this may be excessive when encountering alerts in other settings. Participants concluded that the various alert formats were therefore relevant, and greater detail was important for providing information directly to the health and community services, but excessive detail was problematic if alerts were shared more widely. The summary flyer was thus considered acceptable for use in situations where people did not have time or inclination to filter through comprehensive information included in the detailed alert, and for sharing beyond the clinical setting (e.g. with clients/patients and public audiences).

The inclusion of colour-coded severity indicators was received positively by this group as a potential solution for identifying urgency/severity and minimising ‘alert fatigue’. This group (who had been actively engaged in earlier co-design workshops and feedback sessions) agreed that all three formats were relevant to their workplace needs, and acceptable/useful for a range of situations and audiences. No further feedback was offered when asked what information could be omitted/included in these alerts. When discussing the relevance of different segments of information included (e.g. key contextual information about alerts, clinical management, or harm reduction advice) all felt that the information about substance characteristics, evidence for harms and potential risks were important; while AOD practitioners reported focusing more on harm reduction advice than information about clinical management which was more pertinent to UC practitioners. The following quote highlights variation in what information different workers may pay attention to:

It depends on who this is targeted towards as users will be different to pre-hospital and ED staff. Clinical pathways and recent impacts are very important for health providers. Providing advice on harm reduction and potential effects good and bad will be beneficial to users and AOD workers who deal with them day to day. [UC manager, #42]

One participant noted that engagement with different segments of information and resulting action may depend on the assessment of the relevance of the information and their perceived identity and role:

So maybe I want to get some information as a drug and alcohol manager in that role but then I also may want to get some information that I can forward on to the emergency department’s nurse unit manager. So, I might choose both of those filters of information. [Regional AOD manager, #142].

When asked about ensuing actions after receiving an alert, participants agreed each format (SMS prompt, summary flyer, and detailed alert) included sufficient information to elicit further action such as assessing the relevance of the information to inform decisions about disseminating alert information within and across their networks (e.g. sharing with medical directors, emergency services, raising alerts in workplace meetings, and sharing with clients or patients). They unanimously agreed that the use of hyperlinks and QR codes were useful methods for facilitating information dissemination and providing access to different levels of information for different audience groups.

## Discussion

To the best of our knowledge, this study is the first to explore how to translate information gathered from unregulated drug markets into alerts for use in health and community service settings. Practitioners and managers working in the alcohol and other drugs (AOD) and urgent care (UC) settings contributed to the co-design of three drug alert prototypes. Workers endorsed three final alert prototypes as relevant to their professional needs and acceptable for dissemination to multiple audience groups. Robust co-design conversations revealed important considerations about the tools and mechanisms required for the successful implementation of alerts and local early warning systems (EWS). The implications of these findings are discussed below.

### Establishing the need and context for drug alerts in Victoria

European guidelines for EWS operations suggest that well-informed risk communications provide timely, clear, credible, and consistent evidence-based information necessary to raise awareness of potential threats and offer guidance for more timely and effective responses to drug-related harm at the local, national, and international levels [51]. Participants in this study confirmed that adequately designed alerts were useful tools for achieving three key objectives: (1) promoting widespread awareness of evidence-based intelligence about unexpected market changes and emerging threats; (2) improving workforce responses to drug-related harm in clinical and community settings; and (3) facilitating widespread information exchange (education) across *and* within community and clinical settings about harm reduction/prevention strategies that can (a) support more informed decision-making about substance use among people who use drugs; and (b) reduce the impact/incidence of drug-related harm in the community more broadly. While overdose prevention and behaviour change are often key objectives for harm reduction initiatives, motivations for substance-use and related behaviours are complex and multifaceted. Evaluating the broader social and public health outcomes of public health harm reduction interventions is a complex endeavour that requires sophisticated coordination of multiple indicators at micro-, meso-, and macro-levels [76, 98]. Outcomes will depend on a range of factors that also impact engagement with these systems like the overarching legal, social, and political setting, context of substance/s used, accessibility and practicality of advice offered, and the priorities, motivations, and lived experiences of people who use drugs [45, 52, 99].

We heard from practitioners and managers that communications about unexpected market changes and related harms varied widely across and within healthcare and community organisations. Responses to our initial survey demonstrated that information currently available to AOD and UC workers about changing characteristics of drug markets was not adequate or sufficiently timely to inform their work. Discussions revealed that it was common for these workers to rely on anecdotal, unverified, or ad hoc information about unregulated market supplies to inform their work. While interpersonal communications are useful for disseminating drug-related risk information, participants identified potential for misinformation and/or dilution of the intended message if these are not informed by consistent and reliable information sources, which is consistent with the literature that asserts that communications about unexpected market changes are most reliable when informed by an expert assessment of intelligence across multiple inputs to determine the scope and impact of predicted threats and these are communicated via formal, reliable, and credible channels [44, 51].

In the AOD sector, the perception of role legitimacy and capacity for providing best practice care is impacted by individuals’ attitudes, perceptions, knowledge, and confidence [74]. Some participants of this study (i.e. inexperienced AOD workers) perceived limited knowledge of novel substances to impact their credibility with clients or patients. While AOD specialists have existing knowledge and expertise transferrable to novel and emerging trends, gaps in knowledge (about NPS) have been cited as barriers to delivering effective harm reduction interventions [75, 81]. Key strategies for improving clinical practice at the ‘worker’ level are education, training, and role support—which have been demonstrated to improve workers’ confidence and autonomy in responding to novel substance-use trends [79, 100]. However, it is impossible to develop and deliver timely education about dynamic market changes and the vast number of novel substances available [4, 79, 101]. We argue that alerts for health and community settings can offer timely, lower-barrier methods for disseminating targeted, relevant, and timely expert-informed information to facilitate existing in-service protocols that support workforce capacity for planning for, and responding to, adverse events.

### Alert audiences

Optimising alert protocols so they will be used by the intended audience is vital [48, 70, 89, 102]. Although the scope of this project was to design alerts for healthcare and community service professionals, a recurrent discussion at all co-design and feedback sessions was that people who use drugs are the ultimate alert ‘end users’. The diversity of this population’s needs and preferences was strongly emphasised by participants, and our findings support claims that public health and harm reduction interventions should be informed by participatory, formative evaluations to meet the needs of all affected stakeholders [52, 85, 89, 90, 103].

### Workers’ perceived role/s communicating drug-related risk information

The Australian AOD workforce is comprised of diverse clinical and support roles spanning a vast range of organisations, networks, and programmes [104]. Accordingly, workers in this study identified as having multiple roles when it came to disseminating alert information. AOD practitioners felt that providing education and harm reduction advice was central to their professional practice. UC practitioners also emphasised the importance of not ‘gatekeeping’ alert information from public stakeholders, but they perceived their role in ‘reducing harm’ as maximising patient safety and information exchange to improve clinical responses in acute care settings. AOD-related ‘non-medical emergency’ presentations are increasingly routine work for emergency medicine providers, but some urgent care practitioners maintain stigmatising views about substances use while others have reflected conflicting perspectives about their role in providing non-medical education and support in these situations [105–107].

It has been suggested that policy-sanctioned harm reduction interventions may help to address some of the social and structural determinants of drug-related harm (i.e. stigma) by influencing community attitudes through ‘normalising’ pragmatic harm reduction over abstinence-based approaches [45, 52, 89]. We propose an extension of this might be to expect that alerts embedded into routine clinical practice may help to promote more compassionate, evidence-informed attitudes among clinicians who currently hold limiting views of substance use. One method to do this might be to employ alert ‘champions’ with clearly defined roles for more efficient and effective exchange of reliable evidence-based information to shape workers’ practices, attitudes, and behaviours. Workplace champions have been demonstrated to overcome institutional ‘siloing’ in healthcare, build and leverage professional networks, and cultivate positive learning environments to effect change—although more research on the effectiveness of using champions is necessary to understand their impact in this context [108].

### Challenges faced by workers disseminating risk information

Participants generally considered health and community services as ideal locations for opportunistic ‘risk prevention’ conversations with clients and patients. However, drug information delivered in these settings can be perceived to be slow and inaccurate by some people who use drugs, who tend to rely on peer-to-peer networks as sources of information about drug market quality [69, 70, 72, 73]. Barriers to engaging with harm reduction advice from healthcare providers include imbalanced relationship dynamics, infrequent encounters, clinical priorities that do not align with consumers’ needs (e.g. focusing on unrelatable abstinence-based prevention strategies), and avoidance of discussion about drug quality/purity [69, 72]. Establishing effective therapeutic relationships is key. Compassionate, relatable, non-judgemental information delivered by trusted, well-informed workers can help to facilitate the exchange of credible, consistent, and reliable information about unexpected high-strength, adulterated, or novel substances within the community [52, 69, 71, 72, 102]. Leveraging off existing harm reduction services has been shown to be a low-barrier method for encouraging community engagement with interventions that promote awareness of market adulterations and other perceived threats [19, 48]. Importantly, peer delivery mechanisms seem to be practical and efficient ways to share alert information and harm reduction advice with people who are not actively engaged with health or community services [70, 72, 102].

A key priority for participants in this study was ensuring the involvement of people who use drugs in alert design, planning, dissemination, and evaluation. This may reflect Victoria’s AOD workforce strategy and existing peer workforce that promotes lived/living experience-informed initiatives [104]. There is, however, limited evidence for how peer involvement might impact community engagement with, and responses to, alert information locally. While out of scope for the current study, other Australian research programmes have begun exploring how risk communications impact the motivations and behaviour of people who use drugs locally.^3^ However, as outlined in our companion paper, more research is needed to determine how alert systems can effectively meet the needs of different audiences with diverse substance-use knowledge, experiences, and information needs [86].

### Tailoring alerts to health and community workers’ needs

Our co-design process found that alert design requires careful planning and execution to avoid unintended consequences, maintain credibility with all potential stakeholders, and promote engagement with alert information and advice across a range of different audience groups. We direct readers to Fig. 2 and Table 4 for comprehensive summaries of alert features and important information to include (where possible). Table 2 summarises key factors that will likely impact successful alert design, implementation, and evaluation. In summary, workers specifically wanted access to reliable information from a central, reliable source and low-barrier methods for sharing information.

Our findings support previous alert evaluations that stipulate a need for timely, clear and concise, plain language, evidence-based, and action-oriented information that avoids tokenistic, generic, or unrelatable advice [70, 71]. Similar to findings from evaluations conducted with people who use drugs in the community, healthcare and community workers wanted alerts tailored specifically to the context of the perceived threat and target audience needs, with comprehensive communication strategies that integrate multiple channels/platforms to promote engagement and minimise barriers to accessing and/or sharing information [69, 70, 72, 102]. Participants noted the value of dedicated drug alert applications, alert websites (archive/repository), and social media broadcasts even though these were beyond scope of the current study. While these formats should be considered in future alert system design and evaluation, an important consideration is that some platforms may not be suitable for all audiences (e.g. systems must consider ways to reach people without reliable access to mobile devices, Internet, or email) [109].

Importantly, time-poor AOD and UC practitioners and managers were not interested in repurposing alerts for other audiences. They wanted to be ‘spoon fed’ evidence-based guidance for responding effectively within their role/s (e.g. clear clinical management and/or harm reduction advice). It became clear to us that the alert system (rather than workers) is responsible for creating low-barrier methods that simplify information exchange across and within all audience groups. Participants’ concerns about the risk of diluting critical information or misinterpreting expert-informed advice when translating/repurposing alerts reflect the importance of ensuring drug market intelligence is informed by accurate and reliable evidence-based information [44, 51]. Workshop participants valued the utility of centrally managed alert systems from a single, easily recognisable and trusted alert source. They were concerned that alerts issued by individual organisations or redistributed via other networks could easily be missed with the sheer amount of information they receive daily. The experiences of our participants indicate that the potential for ‘alert fatigue’ (e.g. desensitisation to multiple warnings) is compounded by frequently receiving multiple forms of electronic information and advice from multiple sources. These findings echo problems identified with medical alert systems, where repeated or unnecessary alerts lead to disengagement with information and/or the alert system [110, 111].

### Challenges tailoring alerts to health and community workers’ needs

Alert fatigue is one of five interrelated challenges of drug alert design and dissemination we identified during our co-design process—the implications of these are discussed at length in our companion paper [86]. While there is no ‘one-size-fits-all’ solution to these competing priorities, the following complex challenges must be considered when designing alerts: (1) the need for comprehensive alert information to meet the needs of diverse audiences groups while ensuring clear and concise design; (2) avoiding ‘alert fatigue’ while facilitating timely access to all levels of information required; (3) building trust in alert systems in a ‘prohibition world’; (4) avoiding unintended consequences when issuing alerts; and (5) designing drug alerts for health and community service workers, when alerts are ultimately intended to improve the care provided to people who use drugs.

### The value of coordinated EWS to produce reliable and timely alerts

Given our findings about the benefits of alerts that target the health and community service workforce, and workers’ intentions to share information widely beyond the ‘workplace’ setting, we argue that Victoria has the potential for expanding its existing EWS networks to facilitate the development of alert systems to meet the needs of professional and community audiences. However, co-design conversations revealed many interrelated factors that will likely impact successful implementation of alerts and EWS locally. Workers wanted timely and accessible (and detailed) information delivered from a reliable source, in varied formats across multiple platforms to meet the needs of diverse audience groups. It became apparent that an effective local EWS that supports low-barrier information dissemination (within *and* beyond the workplace setting) requires sufficient resourcing and finding to operate effectively. These findings support learnings from Canada, USA, and Europe that outline key features of successful EWS design: timeliness of information (from detection to resulting public health interventions); system flexibility (adapting to changing legal, social, and political environments; dynamic market trends; audience needs and available inputs); acceptability (stakeholders); inter-disciplinary collaboration (health, harm reduction, law enforcement, etc.); adequate resources (e.g. dedicated core staff, systems, and technology); and ongoing evaluation of reach, engagement, and impact [18, 19, 51]. We argue these learnings from overseas would be useful for informing local emerging EWS.

An increasingly valued feature of contemporary market surveillance and prompt public health responses to unexpected substances (that uniquely prioritises the delivery of timely information directly to people who use drugs) is drug checking—where members of the public submit substances for analysis and receive individualised feedback about results in a range of settings [45, 112–115]. By differentiating between service users’ expectations about samples submitted and analytical results, drug checking offers highly localised, unique and timely insights about unexpected high-strength, novel, or adulterated products in circulation for rapid translation into tailored harm reduction interventions with service users, and can inform local, regional, and cross-jurisdictional alerts/warnings/advisories [45, 116, 117]. Widescale coordinated drug-checking networks that are embedded into broader, public health-oriented EWS may even help to stabilise unregulated markets by reducing local demand for adulterated/contaminated supplies and other substances of concern [65, 117, 118]. However, like for EWS, drug-checking objectives, operations, and outcomes vary widely—often dependent on the social, political, and legal framework in which drug use happens [45, 52, 114]. The viability of drug checking and EWS as public health and harm reduction interventions relies on factors like the quality of surveillance methods (e.g. analysis methods and instruments used) available resources; operational scale, location and setting; data collection, reporting and evaluation methods; the context of substances used/tested; and the motivations, needs, and experiences of target audiences (e.g. people who use drugs) [99, 114, 119, 120].

### Local context for an emerging EWS in Victoria

At the time of writing, public drug alerts in Victoria are predominantly informed by data from EDNAV, which utilises a multidisciplinary network of experts from medicine, forensic sciences, and public health to support holistic interpretation of information shared about substance-related presentations to a network of hospital emergency departments [59, 67]. Hospital settings are useful for sentinel monitoring of substances that have caused acute adverse events in the community and, together with toxicology and clinical information, patient self-reports and actionable descriptions of products consumed can offer insights about the likely presence of unexpected substances in circulation to inform public health alerts [57]. Confirmation of market adulteration is, however, limited when product samples are unavailable for analysis (e.g. in hospital settings after adverse events have occurred) and/or consumers’ expectations about analysed samples are unknown (e.g. forensic seizures) [45].

Based on our findings, we recommend that Victoria should consider developing partnerships across other disciplines to utilise a range of existing surveillance systems (e.g. law enforcement seizures, peer-network intelligence, wastewater analysis, and drug-checking services) to expedite processes for identifying, analysing, and reporting emerging threats in unregulated drug supplies. Features of this proposed EWS could even be ‘scaled-up’ to support more effective, coordinated harm reduction and healthcare interventions during high-risk periods (e.g. festival ‘seasons’) when drug-related harms have been known to spike [26, 62, 121]. However, at the time of writing, local systems are not currently coordinated across disciplines, and there is ‘no active plan for implementation of a drug-checking service’ in Victoria despite coroners repeatedly advocating for the urgent implementation of local drug checking and early and warning networks [32, 34, 35, 54]. While speculative, it is not unreasonable to presume that a widescale, drug-checking-informed EWS may have provided an opportunity for earlier detection and more effective responding to the adulterated MDMA supplies in Victoria that led to a cluster of NBOMe/4-FA hospitalisations and deaths in Melbourne in January 2017 [37].

### Challenges in implementing widescale EWS and drug-checking interventions

International EWS that leverage off existing systems and facilitate multidisciplinary partnerships have been demonstrated as low-barrier methods for establishing successful real-time surveillance for assessing risk and informing public health programmes and policy [19, 48]. However, widescale public health harm reduction responses to unexpected drug harms have traditionally been overshadowed by prioritisation of law enforcement approaches and reactive (rather than preventative) resource allocation [1, 52]. To be attractive to decision-makers, the relative advantage of EWS needs to outweigh risks and cost for them, but with limited evidence of widescale public health benefits (often due to insufficient resources for robust evaluations) these interventions are frequently perceived as costly experiments with questionable/unknown potential for scale-up [45, 89, 99]. In drugs policy—especially in Australia—social, political, and historical factors can hinder the optimal use of evidence in policy and public health practice [53, 122]. For example, drug checking has been successfully trialled in the Australian Capital Territory (ACT) [22, 123], but its implementation in other states and territories remains controversial, where a public debate about drug use is often framed morally or using absolutist characterisations of risk [53, 124, 125]. A low-barrier method for introducing drug checking to inform a local emerging EWS might be to leverage off existing systems within the EDNAV network to allow patients to surrender samples on their person for analytical testing (where possible). Precedence for this has been established by the ACT Investigation of Novel Substances (ACTINOS) group [112]. If successful, this model could be expanded to allow members of the public to submit samples at hospital collection points and retrieve results using unique identifiers, similar to comprehensive drug-checking models that operate in France [112, 119].

### Limitations

Our co-design participants comprised a self-selected sample of AOD and UC workers, who may have had a special interest in novel substances, drug alerts, and related harm reduction interventions. In particular, the perspectives of the two participants working exclusively in UC settings (who did not hold AOD-specific roles in these settings) may not reflect those from non-AOD specialists working across a diverse emergency medicine workforce where patient education about reducing substance-related harms may not be clinicians’ primary focus when responding to adverse events [107]. This was reflected during the testing of the prototypes during the preliminary feedback sessions, where some external stakeholders provided feedback that the detailed alert poster was ‘too much’, challenging the perspectives of co-design participants who were clear on the importance of alert systems including as much information as possible. During the final prototype review, co-design participants offered limited feedback on improving the design and layout of the three alerts presented. We suspect this was partly due to their extensive involvement in the co-design process and feedback sessions, their (strongly expressed) desire for alerts to facilitate information-sharing to avoid ‘withholding’ information or assuming audience knowledge, and acceptance of the summary flyer as a reasonable solution for meeting the needs of different audience groups. In future iterations, prototype testing should also include a wider audience of people who have not participated in the co-design process. In addition, the alert scenario we used was a hypothetical situation based on an alert issued in another jurisdiction [95, 126], as data were not available from Victorian surveillance systems to inform alerts at that time. Uptake and engagement with alert information in future iterations of this multi-phase project may be different in a ‘real alert’ setting evaluated among other members of this workforce, especially given the multiple competing clinical and administrative priorities health and community service workers face daily [127]. Finally, our co-design process was locally informed and therefore reflects the priorities of Victorian workers. Whilst we hope the prototypes developed will have utility for national and global alert systems, alerts should always be tested directly with target audiences locally to ensure suitable design, format, and messaging beyond the context of this study.

### Next steps

This was the first phase of a larger pilot study (Rapid Drug Alerts for Victoria) for eventual implementation, and evaluation of alerts informed by available drug surveillance systems for an emerging EWS in Victoria. The next phase of this study will be to utilise local surveillance systems to implement ‘actual’ alerts based on the alert prototypes designed in this study (based on hypothetical alert scenarios) and evaluate their utility with a similar (but extended) cohort. Further steps include facilitating partnerships between other agencies to contribute to Victoria’s emerging EWS and determining their feasibility for informing local alerts. Our findings have highlighted the need for alerts for health and community workers, but not without including the perspectives of people who use drugs in these evaluations to understand how their needs might reflect and/or differ from the priorities of health and community service workers. Further research should also consider the role of different workers, information sources, and systems design for effectively disseminating alert information within and across clinical and community settings.

## Conclusions

Alerts optimised for use in health and community settings can offer timely, lower-barrier methods for disseminating targeted evidence-based information about unexpected substance and emerging threats to improve workforce knowledge, confidence, and capacity for anticipating, preventing, and responding to drug-related harms. AOD and UC practitioners were identified as having different roles in ‘reducing harm’ that related to their perceived scope of practice (e.g. sharing evidence-based information about harm reduction advice or clinical management advice). However, it is important that alerts targeting this workforce also consider the needs of people who use drugs and public audiences due to workers’ clear intentions to share alert information widely within *and* beyond the workplace setting. In this study, we have highlighted key features of alerts that include the need for easily recognisable, clear and concise (but detailed) information that provides clear harm reduction and clinical management advice, available in multiple formats and delivered by a reliable, single alert source, available on different platforms to meet the needs of diverse clinical, consumer and public audiences. We have also highlighted the need for low-barrier methods to overcome challenges to workers’ engagement with alert information and successful widespread information dissemination.

In the current sociopolitical context of illegal drug markets and frequent stigmatisation of people who use drugs, there are no simple answers to reducing harm associated with dynamically changing market supplies. Comprehensive, multidisciplinary EWS and risk communications (alerts) can, however, play an important role in promoting awareness of drug market intelligence and effective dissemination of information that can empower individuals, organisations, and agencies to respond more effectively to drug-related harm in the community. Alert and EWS systems must, however, be adaptable to the diverse needs of multiple audience groups and changing social and political landscapes and ensure that technologies and systems are sufficiently resourced to meet their objectives and function effectively. Individuals can only achieve so much in terms of disseminating risk information and advice if systems, organisations, and communities are not adequately set up to facilitate EWS and alert system function. Our findings support the need for ensuring that EWS and alert systems engage in comprehensive participatory design and formative evaluation techniques to ensure that public health and harm reduction interventions meet the needs of all relevant stakeholders. Our findings have utility for informing current and future alert systems in settings where the social, cultural, and political context of substance use is similar to that observed in Victoria and across Australia. There is, however, much more work to be done in this space.

## Supplementary Information

**Additional file 1.** Final 'summary flyer' alert prototype.

**Additional file 2.** Final 'detailed poster' alert prototype.

## Data Availability

Qualitative data are not publicly available to protect the privacy of research participants who attended digital workshops. Deidentified data from the brief survey may be made available from the corresponding author upon reasonable request.

## Abbreviations

4-FA: 4-Fluoroamphetamine
AOD: Alcohol and other drugs
CFIR: Consolidated Framework for Implementation Research
COVID-19: Coronavirus disease 2019
EDNAV: Emerging Drugs Network of Victoria
EWS: Early warning system
MDMA: Methylenedioxymethamphetamine
NPS: New psychoactive substances
PDF: Portable document format
PMMA: Paramethoxymethamphetamine
QR: Quick response
SMS: Short message service (mobile phone-based text messages)
UC: Urgent care

## Footnotes

1 ‘Practitioners’ were defined as people working in roles where they engaged directly with people who use drugs in AOD treatment, harm reduction, peer support, outreach services; or people responding to acute drug-related harms in urgent care settings (e.g. doctors, nurses, paramedics, etc.). ‘Managers’ were responsible for allocating resources or staffing for programmes, services, or units in AOD or UC settings.

2 Workshop participants employed across ‘both’ settings were generally AOD practitioners employed within hospital emergency department settings (e.g. AOD nurses/nurse practitioners, and AOD clinical liaison staff).

3 Research underway at the National Drug and Alcohol Research Centre: Risk communication for people who use illicit drugs: establishing guidance on preferences and behavioural responses to drug alerts. Available from https://ndarc.med.unsw.edu.au/project/risk-communication-people-who-use-illicit-drugs-establishing-guidance-preferences-and.
