# Correction: Co‑designing drug alerts for health and community workers for an emerging early warning system in Victoria, Australia

**DOI:** 10.1186/s12954-023-00783-0

**Published:** 2023-04-25

**Authors:** Rita Brien, Isabelle Volpe, Jasmin Grigg, Tom Lyons, Caitlin Hughes, Ginny McKinnon, Stephanie Tzanetis, Sione Crawford, Alan Eade, Nicole Lee, Monica J. Barratt

**Affiliations:** 1 Turning Point, Eastern Health Statewide Services, Richmond, Australia; 2 Monash Addiction Research Centre, Eastern Health Clinical School, Monash University, Melbourne, Australia; 3 Social and Global Studies Centre, RMIT University, Melbourne, Australia; 4 Drug Policy Modelling Program, Social Policy Research Centre, UNSW Sydney, Sydney, Australia; 5 Department of Health, State Government of Victoria, Melbourne, Australia; 6 Law and Commerce, Flinders University, Adelaide, Australia; 7 National Drug and Alcohol Research Centre, UNSW, Sydney, Australia; 8 Harm Reduction Victoria (DanceWize), Melbourne, Australia; 9 CanTEST (Direction Health Services, Pill Testing Australia, Canberra Alliance for Harm Minimisation and Advocacy), Canberra, Australia; 10 Safer Care Victoria, Melbourne, Australia; 11 Department of Paramedicine, Monash University, Melbourne, Australia; 12 360Edge, Melbourne, Australia; 13 National Drug Research Institute, Curtin University, Perth, Australia; 14 Digital Ethnography Research Centre, RMIT University, Melbourne, Australia

**Correction: Harm Reduction Journal (2023) 20:30** 10.1186/s12954-023-00761-6\

Following publication of the original article [1], the affiliation for the author Stephanie Tzanetis has been updated as “CanTEST (Direction Health Services, Pill Testing Australia, Canberra Alliance for Harm Minimisation and Advocacy), Canberra, Australia.”

The original article has been corrected.

