# Supplementary material for: Co-designing drug alerts for health and community workers for an emerging early warning system in Victoria, Australia
Source: Harm Reduct J. 2023 Mar 9;20:30. doi: 10.1186/s12954-023-00761-6 (PMC9995746; doi:10.1186/s12954-023-00761-6)
Supplement: Supplementary file 1 — Additional file 1. Final 'summary flyer' alert prototype. [file 12954_2023_761_MOESM1_ESM.pdf]

DRUG ALERT

Opioid overdose linked to cocaine use

Inner-East & Northern Melbourne April & May 2021

Several unexpected opioid poisonings in people presenting to hospital after reporting cocaine use.

Risk of unexpected opioid overdose may be associated with non-opioid drug use.

Long-acting synthetic opioids identified - overdose symptoms lasted longer than for heroin.

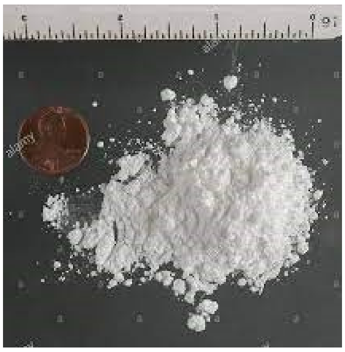

Substances may appear in forms other than white powder.

Drug purity, effects, and appearance always subject to variation.

1 Know signs & symptoms of opioid overdose*

Opioids are depressant drugs that slow down breathing and heart rate. Overdose includes: Unexpected or sudden onset of drowsiness. Loss of consciousness or no response when aroused. Slow, shallow breathing or unusual snoring or gurgling. Blue or grey (ashen) skin, lips or extremities.

**Higher risk of overdose for people with low tolerance to opioid drugs or people using in combination with other depressant drugs (e.g. alcohol, other opioid medications, benzodiazepines, GHB, cannabis).*

2 Know how to respond

HARM REDUCTION ADVICE (scan QR code for more detail)

- Take precautions consuming any type of drugs, be wary of potential unexpected contaminants.
- Carry naloxone (a medicine that reverses opioid overdose).
- In case of opioid overdose: CALL 000 immediately. Administer naloxone if available. Start CPR if not breathing/unresponsive.

CLINICAL MANAGEMENT ADVICE (scan QR code for more detail)

- Suspect opioids poisoning in presentations with signs of opioid overdose.
- Airway management, oxygenation, and ventilation support take precedence over naloxone, if appropriate.
- Due to longer observed effects of synthetic opioids, some cases may require gradual increased, repeated doses of naloxone.

EXAMPLE ONLY

3 Share this information

Share with clinicians, patients, clients, and relevant communities who use drugs.

Scan QR code to sign up and share SMS or email alerts.

SCAN QR CODE FOR MORE INFORMATION ABOUT

REASONS FOR THIS ALERT

OPIOIDS AND RISK OF OVERDOSE

NALOXONE

HARM REDUCTION ADVICE

CLINICAL ADVICE

WHERE TO GET HELP

Drug Alerts Victoria
T: 03 8888 8888
E: vic@drugalerts.org.au
W: www.drugalerts.org.au

REF: 2021-06-001
Issued: 07/06/21
