# Supplementary material for: Co-designing drug alerts for health and community workers for an emerging early warning system in Victoria, Australia
Source: Harm Reduct J. 2023 Mar 9;20:30. doi: 10.1186/s12954-023-00761-6 (PMC9995746; doi:10.1186/s12954-023-00761-6)
Supplement: Supplementary file 2 — Additional file 2. Final 'detailed poster' alert prototype. [file 12954_2023_761_MOESM2_ESM.pdf]

# DRUG ALERT

## Opioid overdose linked to cocaine use

Inner-Eastern & Northern Melbourne April & May 2021

**Drug Alerts Victoria**
**T:** 03 8888 8888
**E:** vic@drugalerts.org.au
**W:** drugalerts.org.au
See website for more information and to subscribe.

### LONG-ACTING SYNTHETIC OPIOID OVERDOSE LINKED WITH COCAINE USE

Several unexpected opioid poisonings in people presenting to hospital after reporting cocaine use across Inner-Eastern & Northern Melbourne (April & May 2021).

Risk of unexpected opioid overdose may be associated with non-opioid drug use.

### SHARE THIS INFORAMTION

Share with networks (colleagues, patients and clients) and relevant stakeholders. Know signs and symptoms of opioid overdose and how to respond, even with non-opioid drug use. Refer to **harm reduction** and **clinical management** advice below.

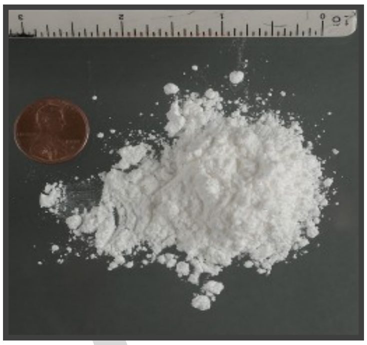

Substances may appear in forms other than white powder.

## BACKGROUND

- 7 people hospitalised for opioid poisoning in Inner-Eastern and Northern Melbourne (April-May 21).
- Patients reported snorting cocaine, but clinical symptoms suggested opioid overdose.
- Blood analysis confirmed presence of strong and long-acting synthetic opioids.
- Previous alerts confirmed synthetic opioids may be present in heroin and other drugs (2020-21).

## DANGERS & RISKS

- Risk of opioid overdose may be associated with non-opioid drug use.
- Duration of synthetic opioid effects is longer than for traditional opioids.
- Higher risk of overdose for people with low opioid tolerance and people not expecting opioid effects.
- Risk of overdose increased if opioids combined with other depressant drugs such as alcohol, other opioid medications, benzodiazepines, GHB, or cannabis.

## SIGNS & SYMPTOMS

### OF OPIOID OVERDOSE

- Unexpected or sudden onset of drowsiness.
- Loss of consciousness or no response when aroused.
- Slow, shallow breathing or unusual snoring or gurgling.
- Blue or grey (ashen) skin, lips or extremities.

**Call 000 immediately and start CPR if person is not breathing.**

## CLINICAL MANAGEMENT (ACUTE RESPONSE)

**Suspect opioids poisoning in presentations with signs of opioid overdose, even if non-opioid use is reported.**

- Airway management, oxygenation, and ventilation support take precedence over naloxone, if appropriate.
- Due to longer observed effects for synthetic opioids, some cases may require gradual increased, repeated doses of naloxone (up to 800mcg+).
- Avoid inducing sudden (precipitated) withdrawal when using naloxone with opioid-dependent people (start with lower doses).
- Consider supplying naloxone to people who use prescription or illicit opioids, and on discharge after opioid poisoning.

## HARM REDUCTION ADVICE

**Opioids are depressant drugs that slow down breathing and heart rate. Take precaution even if consuming other drugs.**

- Know signs and symptoms of opioid overdose and how to respond.
- **Call 000 if a person is unresponsive.**
- People at risk of experiencing or witnessing an opioid overdose should carry naloxone.
- Never use drugs alone. Start low, go slow.
- Drug purity, effects, ingredients and appearance are always subject to variation.
- All drug use comes with risk: Drug Facts.

## GETTING HELP

- **Poisons Information Centre** (acute management advice): **13 11 26**
- **Drug & Alcohol Clinical Advisory Service** (health professionals only): **1800 812 804**
- **Harm Reduction Victoria** (overdose prevention & naloxone training) hrvic.org.au/training
- **24hr confidential drug & alcohol counselling: 1800 888 236** | counsellingonline.org.au

REF: 2021-06-001
Issued: 07/06/21

EXAMPLE ONLY
